# Coronary Artery Stenosis Severity in Patients With Different Coronary Artery Calcium Scores on Coronary Computed Tomography Angiography

**DOI:** 10.7759/cureus.39461

**Published:** 2023-05-25

**Authors:** Kashif A Hashmi, Ammar Akhtar, Farrukh Masood, Shazia Maqbool, Hafiz Muhammad Abdul Kabeer, Jawad Ahmed

**Affiliations:** 1 Cardiology, Chaudhry Pervaiz Elahi Institute of Cardiology, Multan, PAK

**Keywords:** conventional risk factors of cad, coronary artery disease, ct angiography, coronary artery calcium scores, coronary artery stenosis

## Abstract

Background

In this study, we aimed to determine coronary artery stenosis severity in patients with different coronary artery calcium (CAC) scores.

Methodology

A total of 145 patients were included in the study. All patients were given beta-blockers 12 hours and two hours before the test to keep their heart rate between 55 and 65 beats per minute. Computed tomography angiography was done from the pulmonary hilum up to the base of the heart and the patients were asked to hold their breath. The CAC score and stenosis were assessed.

Results

The mean age of the patients was 41.35 ± 4.95 years. In total, 112 (77.24%) patients were male and 33 (22.76%) were female. Regarding the frequency of the CAC score, a score of 0-9 was observed in 43 (29.66%) patients, 10-99 was observed in 55 (37.93%) patients, and 100-400 was observed in 47 (32.41%) patients. The CAC score was 0-9 in 86.4% of patients having normal coronary arteries. Two (5.2%) patients with a CAC score of 100-400 had mild coronary artery stenosis, 11 (32.3%) patients had moderate coronary artery disease, and 33 (66.0%) patients had severe coronary artery disease (p < 0.00001).

Conclusions

There is a strong association between CAC scores and the severity of coronary artery stenosis. A CAC score of zero is associated with a very low risk of having coronary artery stenosis.

## Introduction

Coronary artery disease (CAD) eventually leading to the significant stenosis of the epicardial coronary arteries is a long-term atherosclerotic process. The first presentation of CAD in 50% of patients is acute myocardial infarction [1]. The recent development in imaging techniques has helped considerably in understanding and diagnosing CAD [2]. Coronary computed tomography angiography (CCTA) is one such technique to assess CAD [3,4]. Although CCTA is not comparable to invasive coronary angiography, it has a high sensitivity and specificity of 97.4% and 97.8%, respectively. CCTA is reported in the literature to be 96.5% accurate which is quite impressive [5].

Studies have shown that the coronary artery calcium (CAC) score is an easy tool to risk stratify patients and predict cardiac events [5]. Literature has shown that the CAC score is the better tool to estimate the risk of any major cardiac event as it is more specific and sensitive than the commonly used Framingham risk score [6]. Bhulani et al. have reported that the degree of coronary artery stenosis is directly linked with the CAC score [7].

The CAC score is a relatively cheaper modality. Hence, it can be easily used as a screening tool for CAD. The CAC score can also inform us about the presence and severity of CAD. However, data comparing the extent to which the CAD and CAC score complement each other is very scarce in the South Asian population. Hence, we planned this study to be conducted in our local population to determine the association between the CAC score and CAD on CCTA so that we can start early effective treatment followed by early diagnosis. This will not only lead to a decrease in disease-related morbidity but also improve the quality of life.

## Materials and methods

The descriptive, cross-sectional study was conducted from January 18, 2022, to July 17, 2022, in the Department of Radiology, Chaudhry Pervez Elahi Institute of Cardiology, Multan. The consecutive, non-probability sampling technique was used. Patients with known CAD, acute myocardial infarction, and a history of cerebrovascular accident (confirmed from clinical records and history of the patients) were excluded from this study. Patients of both genders with ages ranging from 20 to 45 years were part of the study. A total of 145 patients were enrolled in the study after obtaining approval from the Ethical Review Board of the hospital and informed consent from the patients (Department of Academic Affairs, Chaudhry Pervaiz Elahi Institute of Cardiology, Multan, approval number: 05). Data regarding age, gender, diabetes, hypertension, obesity, dyslipidemia, and smoking were collected and entered in a proforma. After registration, serum creatinine level was checked and patients were told to abstain from caffeine, tobacco, or any other stimulants for 24 hours before the scan. All patients were given metoprolol 50 mg 12 hours and two hours before the study while ivabradine 5 mg was given to patients who could not take beta-blockers due to any contraindications such as a history of asthma to keep the heart rate between 55 and 65 beats per minute. Intravenous access was obtained with the help of a 20-G cannula. We injected 1 mL/kg of non-ionic iodine contrast containing 350 mg of iodine/mL at a rate of 4 mL/second, followed by 40 mL of normal saline. CT angiography was obtained from the pulmonary hilum up to the base of the heart in a single breath hold. CT angiography was reported by a senior cardiologist with a post-fellowship experience of five years. All the data were entered in the proforma.

Data analysis was performed using SPSS version 23 (IBM Corp., Armonk, NY, USA). Means and standard deviations were calculated for the age of the patients and body mass index (BMI). Frequencies and percentages were computed for gender, obesity, residential status, age groups, smoking, degree of stenosis, calcium score grades, history of diabetes, and hypertension. Effect modifiers such as age, gender, diabetes, obesity, hypertension, smoking, and dyslipidemia were controlled by stratification. Post-stratification, the chi-square test was applied. The chi-square test was also applied to establish a correlation between risk factors and calcium score grading. P-values <0.05 were considered to be significant.

## Results

**Figure 1:**
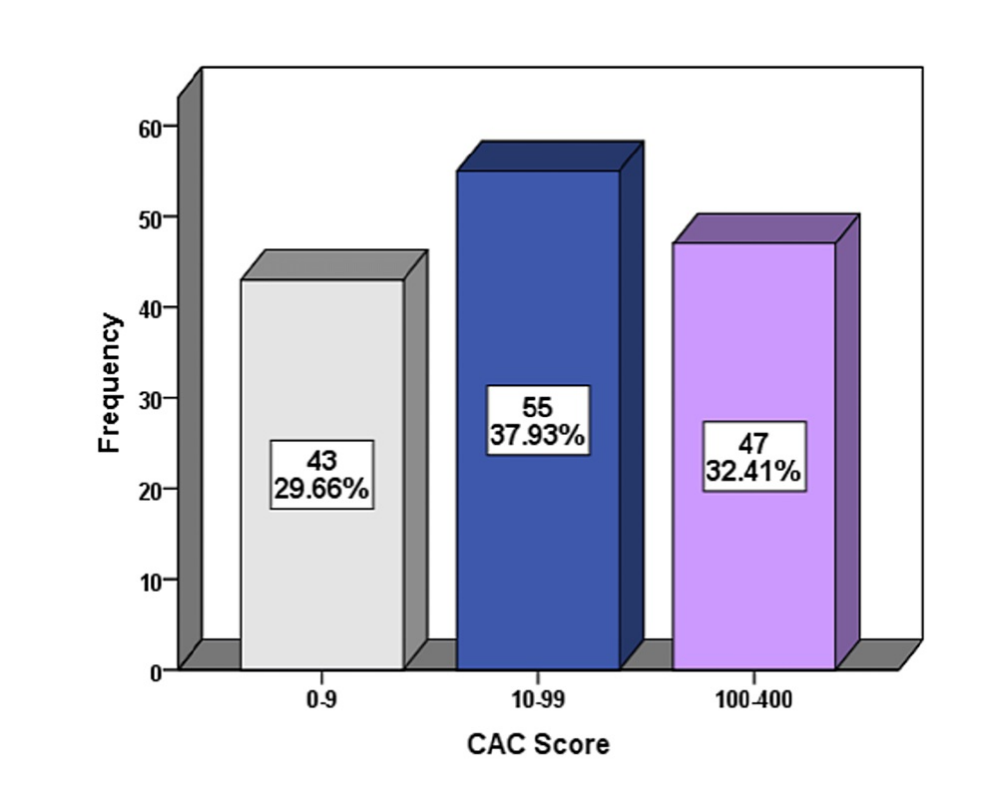
Frequency of CAC scores in our studied population. CAC: coronary artery calcium

A total of 145 patients were included in the study. The mean age of the patients included in this study was 41.35 ± 4.95 years. The mean BMI of the patients included in this study was 26.91 ± 4.95 kg/m^2^. There were 112 (77.24%) male and 33 (22.76%) female patients. There were 77 (53.10%) obese patients, 27 (18.62%) patients were smokers, 14 (9.66%) patients had dyslipidemia, 30 (20.69%) patients were diabetic, and 76 (52.41%) patients were hypertensive. Among the 145 patients, mild stenosis was diagnosed in 39 (26.90%) patients, moderate stenosis in 34 (23.45%) patients, severe stenosis in 50 (34.48%) patients, and normal coronary arteries in 22 (15.17%) patients. Figure 1 shows the frequency and percentages of CAC scores in our study.

Table 1 shows the frequency of conventional risk factors of CAD with each CAC score grading and stratification of risk factors of CAD with the CAC score.

**Table 1:** Frequency and stratification of conventional risk factors of CAD with CAC score. CAD: coronary artery disease; CAC: coronary artery calcium

Risk factors	CAC score			P-value
	0–9	10–99	100–400	
Male	33	39	40	0.233
Female	10	16	7	
Diabetes	10	14	6	0.255
Obesity	21	32	24	0.61
Hypertension	21	26	29	0.21
Smoking	6	9	12	0.31
Dyslipidemia	13	8	3	0.29

Table 2 shows the association of the CAC score with the severity of CAD.

**Table 2:** Association of the CAC score with the severity of coronary artery stenosis. CAD: coronary artery disease; CAC: coronary artery calcium

CAC score classification	Severity of coronary artery stenosis				Pearson correlation coefficient
	Normal (N = 22)	Mild CAD (N=39)	Moderate CAD (N = 34)	Severe CAD (N = 50)	
0–9	19 (86.4%)	19 (48.7%)	4 (11.8%)	1 (2.0%)	0.128
10–99	2 (9.1%)	18(46.1%)	19(55.9%)	16 (32.0%)	
100–400	1 (4.5%)	2 (5.2%)	11 (32.3%)	33 (66.0%)	

## Discussion

The literature has shown that the CAC score is better in predicting cardiovascular disease and mortality compared to the traditional risk factors. Moreover, the absence of CAC projected extremely low mortality rates [8]. CCTA has been shown to be a better and less invasive imaging modality to detect CAC stenosis and atherosclerotic CAD.

Our study has shown that the CAC score is associated with the degree of coronary stenosis. We found a positive relationship between the CAC score and the severity of CAD, although a strong association was not established due to the small sample size. Although different types of CAC scoring systems exist, the Agatston scoring system is the one that is widely used [9], and we have also used the Agatston scoring system in this study. Several studies have shown that in patients with symptoms of chest pain, a CAC score of zero has high sensitivity and a negative predictive value for excluding obstructive CAD [10]. We found that patients with normal coronaries had CAC scores ranging between 0 and 9, and the majority of patients with severe CAD had CAC scores ranging between 100 and 400. The results of our study are also supported by the study conducted by Bhulani et al. They reported mild stenosis in 3.3%, moderate in 2.3%, and severe in 5.3% when the CAC score was 0-9. When the CAC score was 10-99, they reported mild stenosis in 73.6%, moderate in 7.4%, and severe in 1.7%. When the CAC score was 100-400, they reported mild stenosis in 16.5%, moderate in 54.4%, and severe in 29.1%. When the CAC score was more than 400, mild stenosis was 10.5%, moderate stenosis was 15.8%, and severe stenosis was 73.7% [7]. This shows that there is a strong association between the CAC score and the severity of CAD.

We found that a majority of males had CAC scores of 100-400. Similarly, obese, diabetic, and hypertensive patients were found to have CAC scores of 100-400. However, no significant association was found between the major conventional risk factors of CAD and CAC score. Church et al. conducted a study on conventional risk factors and their association with CAC score and reported hypertension in 9% of patients having a CAC score <100 and in 16% of patients having a CAC score ≥100, smoking history in 5% of patients having a CAC score <100 and in 5% patients having a CAC score ≥100, and diabetes mellitus in 5% patients having a CAC score <100 and in 9% patients having a CAC score ≥100. [11] Our study is in contradiction with the study conducted in Iran by Yazdi et al. We were unable to establish a strong association between conventional risk factors and CAC score, but Yazdi et al. revealed that a non-zero CAC score is strongly associated with the conventional risk factors of ischemic heart disease [12].

Our study has some limitations. Our study has a small sample size, is single-centered, and is a one-time study. However, to our knowledge, this is the first study conducted among the local population. There is a need to conduct such studies at a larger level with a larger sample size and proper follow-up. More randomized control trials with larger sample sizes should be conducted to determine the association of CAC scores with conventional risk factors of CAD in our local population.

## Conclusions

There is an association between the CAC score and the severity of CAD. A CAC score of zero is associated with a very low risk of having coronary artery stenosis. Severe CAD is associated with an increased CAC score. The CAC score is an independent predictor of severe CAD. The CAC score is an easy, non-invasive, and cheap way to predict CAD. It can also be used to start primary prevention for atherosclerotic cardiovascular disease in high-risk patients.
